# Chemical and mechanical activation of resident cardiac macrophages in the living myocardial slice ex vivo model

**DOI:** 10.1007/s00395-022-00971-2

**Published:** 2022-11-30

**Authors:** F. J. G. Waleczek, M. Sansonetti, K. Xiao, M. Jung, S. Mitzka, A. Dendorfer, N. Weber, F. Perbellini, T. Thum

**Affiliations:** 1 Institute of Molecular and Translational Therapeutic Strategies (IMTTS), Hannover Medical School, Carl-Neuberg Straße 1, 30625 Hannover, Germany; 2 Fraunhofer Institute ITEM, Nikolai-Fuchs-Straße 1, 30625 Hannover, Germany; 3 Walter-Brendel-Centre of Experimental Medicine, University Hospital, Ludwig-Maximilians-University München, Marchioninistraße 27, 81377 Munich, Germany

**Keywords:** Myocardial slices, Resident cardiac macrophages, Inflammation, Mechanical load

## Abstract

Resident cardiac macrophages (rcMACs) are among the most abundant immune cells in the heart. Plasticity and activation are hallmarks of rcMACs in response to changes in the microenvironment, which is essential for in vitro experimentation. The in vivo investigation is confounded by the infiltration of other cells hindering direct studies of rcMACs. As a tool to investigate rcMACs, we applied the ex vivo model of living myocardial slices (LMS). LMS are ultrathin ex vivo multicellular cardiac preparations in which the circulatory network is interrupted. The absence of infiltration in this model enables the investigation of the rcMACs response to immunomodulatory and mechanical stimulations. Such conditions were generated by applying interferon-gamma (IFN-γ) or interleukine-4 (IL-4) and altering the preload of cultured LMS, respectively. The immunomodulatory stimulation of the LMS induced alterations of the gene expression pattern without affecting tissue contractility. Following 24 h culture, low input RNA sequencing of rcMACs isolated from LMS was used for gene ontology analysis. Reducing the tissue stretch (unloading) of LMS altered the gene ontology clusters of isolated rcMACs with intermediate semantic similarity to IFN-γ triggered reaction. Through the overlap of genes affected by IFN-γ and unloading, we identified *Allograft inflammatory factor 1* (*AIF-1*) as a potential marker gene for inflammation of rcMACs as significantly altered in whole immunomodulated LMS. MicroRNAs associated with the transcriptomic changes of rcMACs in unloaded LMS were identified in silico. Here, we demonstrate the approach of LMS to understand load-triggered cardiac inflammation and, thus, identify potential translationally important therapeutic targets.

**Supplementary Information:**

The online version contains supplementary material available at 10.1007/s00395-022-00971-2.

## Introduction

Cardiac tissue inflammation is a complex pathophysiological process that involves multiple cell types. The reaction and resolution of cardiac inflammation depend on the cross-talk between cardiomyocytes, cardiac fibroblasts, endothelial cells, and immune effector cells [40, 54]. Alterations in inter-cellular interactions can result in excessive extracellular matrix deposition, tissue remodeling [14, 54], maladaptive cardiac function and increased arrhythmic tendencies [32]. These conditions can eventually lead to heart failure [15]. Besides the role in homeostasis and efferocytosis [3, 48], the contribution of rcMACs during disease state has not been well appreciated. Several recent studies have highlighted their crucial function in tissue repair not only during steady-state, but also in damaged hearts [18, 51]. In line, the depletion of this cell type results in impaired cardiac regeneration [9]. After interacting with immunomodulatory compounds like interferon-gamma (IFN-γ) or interleukin-4 (IL-4), macrophages classically undergo polarization and transition towards an activated state [26, 30]. Macrophages can alter the microenvironment of other cardiac cells [28], play a pro-fibrotic role by stimulating other cells to secrete collagen [19], and influence cardiomyocytes fractional release of Ca^2+^ [16]. A better understanding of rcMACs’ significance and the modalities that regulate their activation attract the scientific community’s interest for their use in discovering novel therapeutic strategies.

To date several factors have limited the investigation of rcMACs. One factor is their relatively small number, estimated to be approximately 7.5% of the total cardiac cells [12]. The second factor is the rapid de-differentiation of rcMACs during in vitro culture and consecutive alterations of their response to various chemical and mechanical cues [29]. This approach, however, has the advantage of a precise cytokine supplementation to the culture media. In vivo, deregulated cytokine production and cytokine injections lead to dynamic recruitment and infiltration of circulating inflammatory cells [12], modulating rcMACs response. This process starts with the early infiltration of neutrophilic granulocytes, followed by a second infiltration wave made of bone marrow-derived macrophages [21, 61]. These factors impede the direct investigation of rcMACs role in health and disease. Novel cardiac models for unbiased studies of rcMACs are required. One alternative to the current in vivo [27] or in vitro models are living myocardial slices (LMS) [56]. In LMS, rcMACs can be cultivated in the multicellular context of the myocardium and the accurate cytokine supplementation allows a precise investigation of rcMACs response to the chemical stimulation.

LMS are ultrathin (100–400 µm) cardiac tissue sections proposed as an innovative multicellular ex vivo model for advanced cardiac research [58]. The rapid development of novel ex vivo culture methods and devices capable of delaying structural and functional changes associated with chronic ex vivo culture allows scientists to address new scientific questions and investigate specific cell types while cultured in the context of the cardiac multicellular environment. LMS have already been used for drug testing [34, 41], to investigate cardiac fibroblasts [41] and the transmural electrophysiological differences of the myocardium [44]. Alterations of the mechanical load have also been applied and studied using this ex vivo platform [43, 44, 57]. To generate LMS, the heart is removed from the body, and LMS are prepared with a high-precision vibratome. This process disconnects the tissue from the vascular network, implying that new inflammatory cells can not migrate and infiltrate the tissue. In this study, we took benefit of this multicellular platform to investigate the rcMAC’ immunomodulatory response upon defined cytokine supplementation and compare the chemical rcMAC activation with the effects induced by conditions of unload or overload to identify targets that could be tested for therapeutic applications.

## Materials and methods

### Living myocardial slice preparation and functional evaluation

The heart samples were obtained from 8- to 12-week-old male Sprague Dawley rats. All animal experiments were registered and approved under §4 Tierschutzgesetz by the LAVES (Niedersächsisches Landesamt für Verbraucherschutz und Lebensmittelsicherheit) of Lower Saxony under registration number 2017/117. The animals were housed under specific pathogenic-free conditions in the central animal facility of the Medical School Hanover (MHH/ZTL). They were fed ad libitum in controlled humidity, temperature, and available lighting conditions. In this study, LMS were prepared as described in previous publications [58]. Briefly, modified Tyrode’s slicing solution [58] containing 30 mM 2,3-butanedione monoxime (BDM), 140 mM sodium chloride, 12 mM potassium chloride, 0.33 mM Sodium phosphate monobasic dihydrate, 10 mM anhydrous D( +)-glucose, 10 mM HEPES, 1 mM magnesium dichloride, 0.9 mM calcium dichloride, dissolved in water and adjusted to pH 7.4, was prepared. The solution used for cell isolation and tissue processing was filtered with a 0.2 µm bottle-top vacuum filter, and 100 IU of heparin sodium was added prior to heart explantation. The animals were anesthetized with 4% isoflurane at 1L/min O_2_ until the postural and interdigital reflex expired_,_ and after cervical dislocation, the heart was quickly removed [58]. LMS were prepared with high-precision vibrating microtomes (Model 7000SMZ-2, Campden Instruments LTD) fitted with a temperature-controlled tissue bath filled with 4 °C cold modified Tyrode’s solution. The Z-alignment of the blade vibration was calibrated to be < 1 µm before each experiment. With a vibration amplitude of 2 mm, a blade frequency of 80 Hz, and a cut speed of 0.07 mm/s, 300 µm thick slices were generated from rat myocardium. Slices designated for a prolonged ex vivo culture were trimmed to approximately 6.5 mm × 6.5 mm using a razor blade, accordingly to the cardiac muscle fiber alignment as previously described [58]. 3D-printed plastic rings (polylactic acid) were attached perpendicular to the fiber alignment with surgical histoacryl glue (Braun GmbH). The LMS were cultured in cardiac slice culture media which was made up with M199 with 1:1000 ITS 100X liquid media supplement (Sigma-Aldrich) and 3% Penicillin/Streptomycin (Gibco). The LMS were cultured ex vivo with the biomimetic culture system described by Fischer et al. [6]. This culture device allows for constant monitoring of LMS contraction (acquisition rate of 400 Hz), which provides data for both, force production and contraction kinetics. For this study, the parameters considered were force (amplitude), time to peak and time to 90% of relaxation. LabChart (ADInstruments Ltd.) was used for this analysis. For chronic experiments in which the tissue preload was modified, the target load applied was based on the correlation of relative stretch and sarcomere length (SL) described by Watson et al. [57]. With no external forces applied to freshly prepared slices (unloaded condition = 0% stretch), sarcomeres adopted a resting length of 1.8 µm (SL1.8). The physiological load (SL = 2.1 µm, SL2.1) corresponded to 12.6% stretch, and overload (SL2.4) corresponded to 27% distension from resting length [57]. To investigate rcMAC response to pro- and anti-inflammatory compounds, recombinant rat IL-4 (20 ng/ml, Peprotech) or recombinant rat IFN-γ (20 ng/ml, Peprotech) were added to the culture medium. The tissue was electrically paced at 1 Hz. The culture device was kept in an incubator at 37 °C, with 5% CO_2_. Contraction recordings were acquired over 24 h and analyzed at 4 h intervals using Clampfit10.7 (Molecular Devices, LLC) or LabChart 7 Pro (ADInstruments Ltd).

### Immunofluorescence staining and microscopy

LMS were fixed in 4% paraformaldehyde (PFA) for 20 min to image macrophages in whole myocardial slices. Fixed LMS were stored in phosphate-buffered saline (PBS) at 4 °C. LMS staining and imaging were performed as previously described [41]. Briefly, the slices were permeabilized in 1% Triton X (Roth) diluted in PBS for 4 h at room temperature (RT). LMS were incubated for 4 h at RT in a blocking solution prepared with 10% fetal bovine serum (FBS, Santa Cruz), 5% bovine serum albumin (Sigma) and 10% horse serum (Thermo Fisher Scientific) in PBS. The primary antibodies were diluted in PBS, and samples were incubated at 4 °C overnight. LMS were washed three times for 30 min with PBS and incubated with the secondary antibody solution for 3 h at RT. LMS were labeled with Hoechst33342 diluted 1:1000 in PBS for 15 min, and the samples were washed three times for the duration of 15 min. Used antibodies and concentrations are stated in Table 1. Image acquisition was performed with Leica Inverted 3 confocal microscope using 20 × magnification, 1.00 Zoom. Z-series images were acquired and processed using Fiji [49]. The various cell types, positive for DAPI, Vimentin (VIM), CD163, and CD45 were counted. For each picture, at least 150 DAPI positive cells were counted.

**Table 1:** Antibodies

Antibody	Manufacturer	Antibody number	Concentration	RRID
Rabbit Anti-CD45	Abcam	ab10558	1:250	RRID:AB_442810
Mouse Anti-CD11b/c	Abcam	ab1211	1:250	RRID:AB_442947
Rabbit Anti-CD163	Abcam	ab182422	1:250	RRID:AB_2753196
Chicken Anti-Vimentin	Invitrogen	PA1-10003	1:2000	RRID:AB_2216267
Hoechst33342	Thermo Fischer	H3570	1:1000	RRID:AB_106267
Anti-Rabbit Alexa Fluor 488	Thermo Fischer	R37118	1:2000	RRID:AB_2556546
Anti-Mouse Alexa Fluor 594	Thermo Fischer	A-11005	1:2000	RRID:AB_2534073
Anti-Chicken Alexa Fluor 647	Thermo Fischer	A-11039	1:2000	RRID:AB_142924

Isolated rcMACS (for isolation procedure refer to “Antibody-mediated cell isolation” section) were seeded in 6-well plates (Sarstedt) on 5 mm round glass coverslips (VWR) coated with 0.1% gelatin (Sigma) in 1 ml M199 with 3% P/S and 1:1000 ITS. After 24 h, the cells were fixated with 4% PFA and stained following the same protocol as for LMS. Nikon epifluorescent microscope with 10 × magnification was used to acquire the images. The cells were labeled for CD11b/c and CD163 and the total cell number was identified by the nuclei being stained by Hoechst 33342. The ratio of positive cells is expressed in respect to the total nuclei number.

### Enzymatic slice digestion

Fresh or cultured LMS were enzymatically digested in a two-step protocol using Collagenase A (> 0.15 U/mg, Sigma-Aldrich) diluted in modified Tyrode’s solution. The first solution had 4.9 mg of enzyme dissolved in 3.5 ml of Tyrode’s solution. After 15 min of incubation time on the rocker at 37 °C, the supernatant was removed. The remaining tissue fragments were incubated for 20 min in solution B, made with 4.9 mg Collagenase A dissolved in 5.2 ml modified Tyrode’s solution. The isolated cells were transferred into Dulbecco’s Modified Eagle Medium with 4.5 g/l glucose (DMEM, Gibco) with the addition of 10% FBS and 4% 20 mM ethylenediaminetetraacetate (EDTA, Gibco). The solution containing the freshly isolated cells was filtered with a 40 µm cell filter to remove large tissue fragments, then centrifuged for 5 min at 300 *g* and 4 °C. The supernatant was then removed, and red blood cell lysis was performed with 300 µl 10 × MACS red cell lysis solution (Miltenyi Biotec) diluted in 2.7 ml pure distilled water (Invitrogen) with an incubation time of 2 min. After centrifugation for 5 min, 300 *g* and 4 °C, the blood cell lysis solution was removed. The cell pellet was resuspended with MACS rinsing solution (Miltenyi Biotec) prepared with 10% FBS. The cells were counted and centrifuged at 300 *g*, 4 °C for 5 min and resuspended in M199 with 1:1000 ITS and 3% Penicillin/Streptomycin.

### Antibody-mediated cell isolation

Cell isolation from freshly prepared LMS or from 24 h ex vivo cultured LMS were performed using CD11b/c anti-rat MACS antibody beads, and MS MACS columns fitted with a 20 µm MACS pre-separation filter. These cells were used, respectively, for gene expression analysis of rcMACs seeded onto coverslips and for RNA-seq experiments. In addition, the columns were fitted into a magnetic MACS 8-column MultiStand (all material from Miltenyi Biotec). The cell isolation was performed according to the manufacturer’s recommendation, resulting in 1 ml of CD11b/c positive cells and 2.5 ml of CD11b/c negative cells. Up to 1.5*10^5^ cells were seeded on 0.1% gelatin-coated 6-well plates (Sarstedt) and stimulated with recombinant rat IL-4, 20 ng/ml (Peprotech) or recombinant rat IFN-γ, 20 ng/ml. Plates were incubated at 37 °C, with 5% CO_2_ and 18% O_2_ (C 170, Binder GmbH) for up to 48 h.

### RNA extraction

Whole LMS were homogenized for 2 × 40 s at 5500 RPM in tubes with six 2.4 mm zinc-ceramic balls (Precellys 24) in 700 µl of QIAzol (Qiagen). Cell suspension samples were prepared for RNA extraction with 700 µl of QIAzol lysis reagent. RNA extraction resulted in 30 µl of eluted RNA for whole tissue slices (RNAeasykit Mini, Qiagen) or in 14 µl of eluted RNA for isolated cells (RNAeasykit Micro, Qiagen) with expected low RNA yield. Quantity and purity were measured with a micro-volume plate reading photometer (Synergy HTX/Take3, BioTeK), and samples were stored at − 80 °C.

### qRT-PCR analysis

Reverse transcription was performed with 125 ng equals of extracted RNA in a total of 13 µl nuclease-free water. A thermocycler performed the cDNA synthesis (TProfessional Trio, Biometra Ltd.) with a reaction containing 4 µl iScript Reaction Mix (Bio-Rad), 2 µl oligo-dT Primer and 1 µl iScript reverse transcriptase (Bio-Rad). The transcription protocol times were 90 min at 42 °C, 5 min at 85 °C and a hold period at 8 °C resulting in 20 µl of complementary DNA (cDNA). The transcribed 1:3 prediluted cDNA was then analyzed with 5 µl SYBR Green reaction (Bio-Rad), 0.025 µl iQ SYBR 20 × Precision Blue RT-PCR Dye, 0.05 µl ROX 1:50 and 10 µM of specific high purity salt-free forward and reverse primers (Eurofins) diluted in 2.425 µl of nuclease-free water and with 2 µl cDNA per reaction. The qPCR reaction was done in 45 cycles (95 °C for 3 min pre-dissociation, 95 °C for 15 s, 60 °C for 30 s and 72 °C for 40 s). Primer-specific amplification was confirmed with melt curve analysis and controls with and without reverse transcriptase added, compared to water. Primer sequences are found in Table 2. The changes in gene expression were calculated using the ΔΔ-CT method and expressed as normalized to the mean of two reference genes (*GAPDH*/*ACTB*).

**Table 2:** Primer sequences

Target	Forward	Reverse
*CD68*	5-AATCAACCTACCAGCCCCTC-3	3-CTTACCAGGAGTGCGTACCA-5
*CD45*	5-AGACAGAGGGCAAAGGAGAC-3	3-CCTTGCCCTGTGACAAAGAC-5
*CD11b*	5-GGGGCCCCTCATCACTATG-3	3-ACATCTCCCAGCACTGTCAA-5
*ARG1*	5-ACCTGCTGGGAAGGAAGAAAAG-3	3-CTGTAAGATAGGCCTCCCACAA-5
*NOS2*	5-CGCTGGTTTGAAACTTCTCAGC-3	3-GTTGACTAGGCTAACAAGACCC-5
*MMP9*	5-TTACATCGGAGGACACCACC-3	3-GAGGGATCATCTCGGCTACC-5
*MMP2*	5-TACTAGGACCTGCAAGCACC-3	3-CAATCGTGCCTCCATCCTTG-5
*DDR2*	5-AACTACAGTCGGGATGGCAA-3	3-ACGTTCATGGAGTGGTCAGT-5
*POSTN*	5-GTTCCTGTGTGACGTTGACC-3	3-CGGGGCAGCATTCATATAGC-5
*ENG*	5-AACTTAGCCCTGCACCCTAG-3	3-CGATGCTGTGGTTGGTACTG-5
*Ly6C*	5-TTTCTCTGTGCACCCCTTCT-3	3-GGAAACTTGGGGCAGATTGG-5
*MerTK*	5-ATGCTCTTCTGGCCTCTGAG-3	3-CTCCCCTAGCCTCTGTGTTT-5
*IL6*	5-CTCTCCGCAAGAGACTTCCA-3	3-AGTCTCCTCTCCGGACTTGT-5
*DDR2*	5-AACTACAGTCGGGATGGCAA-3	3-ACGTTCATGGAGTGGTCAGT-5
*COL4A1*	5-GCAGGAGGGGAAGATCACTA-3	3-TATTAGCGGTCTGTGTGGCA-5
*COL1A1*	5-GAGTTTCCGTGCCTGGCCCC-3	3-ACCTCGGGGACCCATCTGGC-5
*COL3A1*	5-GAAACCCCAGCAAAACAAAA-3	3-TATTGGTGGGTGAAACAGCA-5
*AIF-1*	5-GATCAACAAGCACTTCCTCG-3	3-GACATAATATCGATATCTCC-5
*ACTB*	5-CTGAGGAGCACCCTGTGCTG-3	3-CCAGAGGCATACAGGGACAA-5
*GAPDH*	5-GAAGGGCTCATGACCACAGT-3	3-GGATGCAGGGATGATGTTCT-5

### RNA-seq and data analysis

1 ng of total RNA was used for library preparation with the ‘SMARTer Stranded Total RNA-Seq Kit v2—Pico Input Mammalian’ (#634413; Takara/Clontech) according to conditions recommended in the user manual #063017. Generated libraries were barcoded by a dual indexing approach and were finally amplified with 12 cycles of PCR. Fragment length distribution of generated libraries was monitored using ‘Bioanalyzer High Sensitivity DNA Assay’ (5067–4626; Agilent Technologies). The libraries were quantified using the ‘Qubit^®^ dsDNA HS Assay Kit’ (Q32854; Thermo Fisher Scientific). Equal molar amounts of twenty libraries were pooled for a common sequencing run. Accordingly, each analyzed library constitutes 5% of the overall flowcell capacity. According to the Denature and Dilute Libraries Guide, the library pool was denatured with NaOH and diluted to 2 pM (Document # 15,048,776 v02; Illumina). The denatured pool (1.3 ml) was loaded on an Illumina NextSeq 550 sequencer using a High Output Flowcell for single reads (20,024,906; Illumina). With the following settings, sequencing was performed: Sequence reads 1 and 2 with 38 bases each; Index reads 1 and 2 with eight bases. Using bcl2fastq Conversion Software version v2.20.0.422 (Illumina), BCL files were converted to FASTQ files. Raw data processing was conducted using nfcore/rnaseq (version 1.4.2), a bioinformatics best-practice analysis pipeline used for RNA sequencing data at the National Genomics Infrastructure SciLifeLab Stockholm, Sweden. The pipeline uses Nextflow, a bioinformatics workflow tool which pre-processes raw data from FastQ inputs, aligns the reads and performs extensive quality control on the results. The genome reference and annotation data were taken from Illumina’s iGenomes platform (http://igenomes.illumina.com.s3-website-us-east-1.amazonaws.com/Rattus_norvegicus/Ensembl/Rnor_6.0/Rattus_norvegicus_Ensembl_Rnor_6.0.tar.gz). Normalization and differential expression analysis were performed with DESeq2 (Galaxy Tool Version 2.11.40.2). The default settings were used except for “Output normalized counts table”, “Turn off outliers replacement”, “Turn off outliers filtering”, and “Turn off independent filtering”, all of which were set to “True”. Gene set enrichment analysis (GSEA) was performed using log2(FC) values of significantly deregulated genes (*p* < 0.05) with the WEB-based GEne SeT AnaLysis Toolkit (RRID:SCR_006786) [25]. The organism of interest was “Rattus norvegicus”, and the functional database was “geneontology” with “biological processes nonReduntent” or “network” with “miRNA target”. No advanced parameter was changed. The result of the miRNA target prediction was clustered with the “Affinity propagation” option.

### Statistical analysis

Acquired data were processed with GraphPad Prism 9.1.0 (RRID:SCR_002798, GraphPad Software Inc.). Data are shown as mean ± SEM, if not otherwise stated. Data were first tested for normal distribution. A statistical Student’s *t*-test, one-way analysis of variance (ANOVA) with post hoc Tukey’s or Dunnett’s for multiple comparisons was performed, if normality was assumed. If normality was not assumed, Mann–Whitney or Kruskal–Wallis tests were used. For the RNA-seq Data, multiple *t*-tests with FDR < 0.05 were performed comparing subgroups. Pathway analysis *p*-values were adjusted with FDR < 0.05 correction. *P*-values ≤ 0.05 were considered statistically significant (*p* ≤ 0.05*, *p* ≤ 0.01**, *p* ≤ 0.001***, *p* ≤ 0.0001****). Venn diagrams were drawn using free online software [17]. G-SESAME online tool (RRID:SCR_005816) was used to measure the semantic similarity of ranked GO-gene sets using the AIC method [55].

## Results

**Fig. 1:**
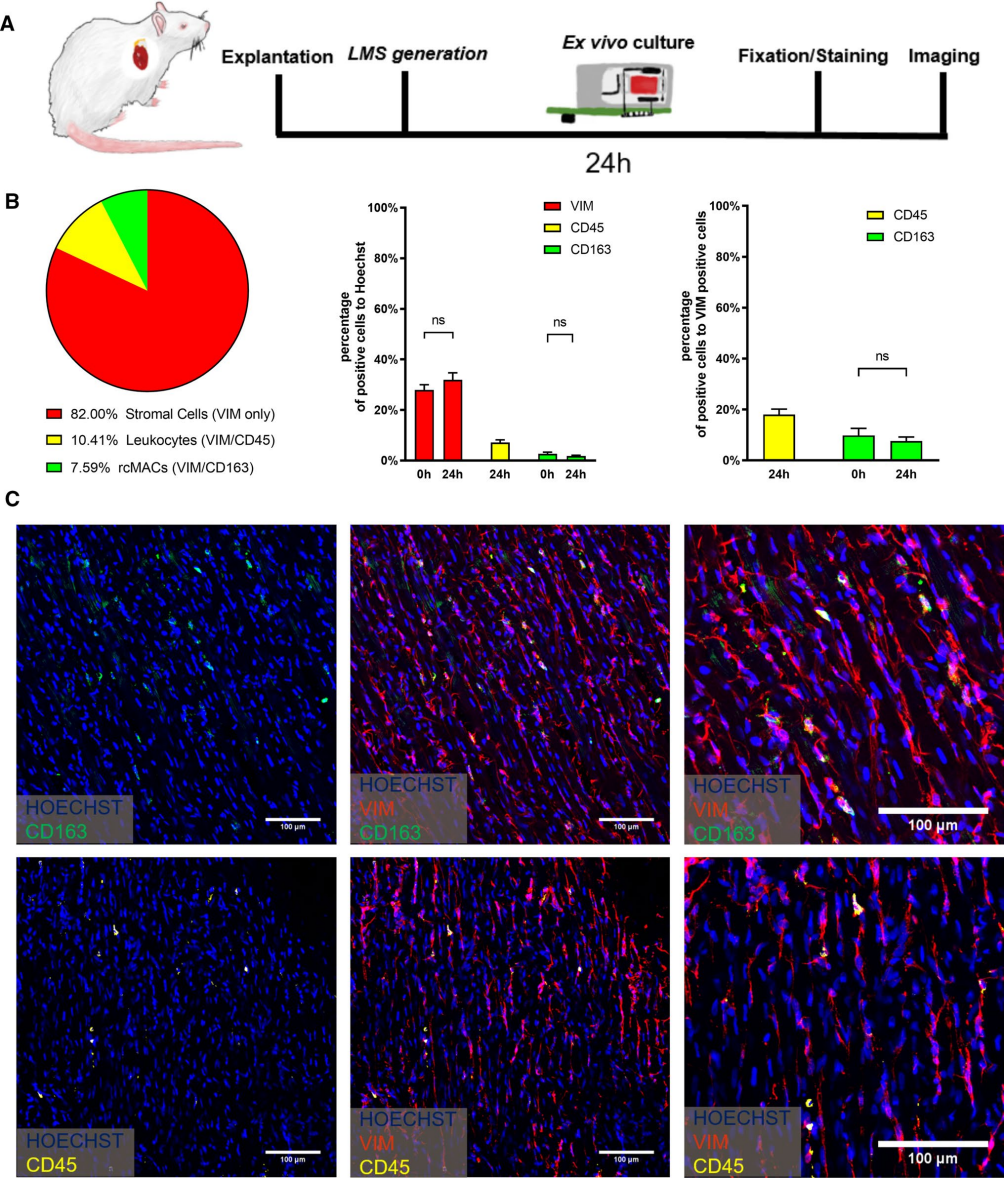
**A** LMS were cultured ex vivo for 24 h in biomimetic culture chambers and electrically paced at 1 Hz. They were fixed, stained and imaged by confocal microscopy. **B** Pie chart shows (left) the stromal cell composition of fresh rat LMS and after 24 h of ex vivo biomimetic culture. Leukocytes and rcMACs made up 10.4 ± 4.8% and 7.6 ± 3.5% (mean ± SD), respectively, and the remaining cardiac stromal cells were exclusively VIM-positive (82.0%). The comparison of 0 h and 24 h (prepared from *n* = 2 or *n* = 5 animals, respectively) LMS did not show a difference in the amount of VIM and CD163 positive cells. **C** Representative average Z-stack projected pictures of LMS stained with Hoechst33342 (blue), VIM (red), CD45 (yellow) or CD163 (green). Nineteen pictures from 5 myocardial slice sections produced out of five animals were processed. A total of 5702 cells were counted

In this study, we identified and quantified rcMACs in rat ex vivo cultured myocardium. LMS were prepared as previously described [58] and cultured ex vivo in biomimetic culture chambers for 24 h [6]. This culture system allowed constant monitoring of tissue contractility combined with electrical pacing (1 Hz) and medium agitation applied by a rocking motor performing 50 oscillations per minute. The LMS were fixed in 4% paraformaldehyde and stained to identify and count the various cell types (Fig. 1A). The cardiac cellular composition has been extensively described in the literature [41, 42]. Therefore, we focused solely on the cardiac immune cell population and rcMACs. Hoechst33342 was used to determine the cell nuclei and vimentin (VIM) to identify stromal cells [7]. CD45 antibody staining revealed hematopoietic cells, whereas ED2/CD163 is a cell marker exclusively expressed by mature tissue macrophages [45]. The quantification showed that after 24 h in culture, 31.9% of all counted cells stained for VIM, hematopoietic cells (CD45 +) were 18% and adult tissue macrophages (CD163 +) 7.6% of the total stromal cell population (Fig. 1B, C). The fraction of CD163-positive cells remained preserved over 24 h of LMS culture (Fig. 1B, right). We conclude that LMS contain a small population of rcMACs, and their number is preserved during ex vivo culture.

**Fig. 2:**
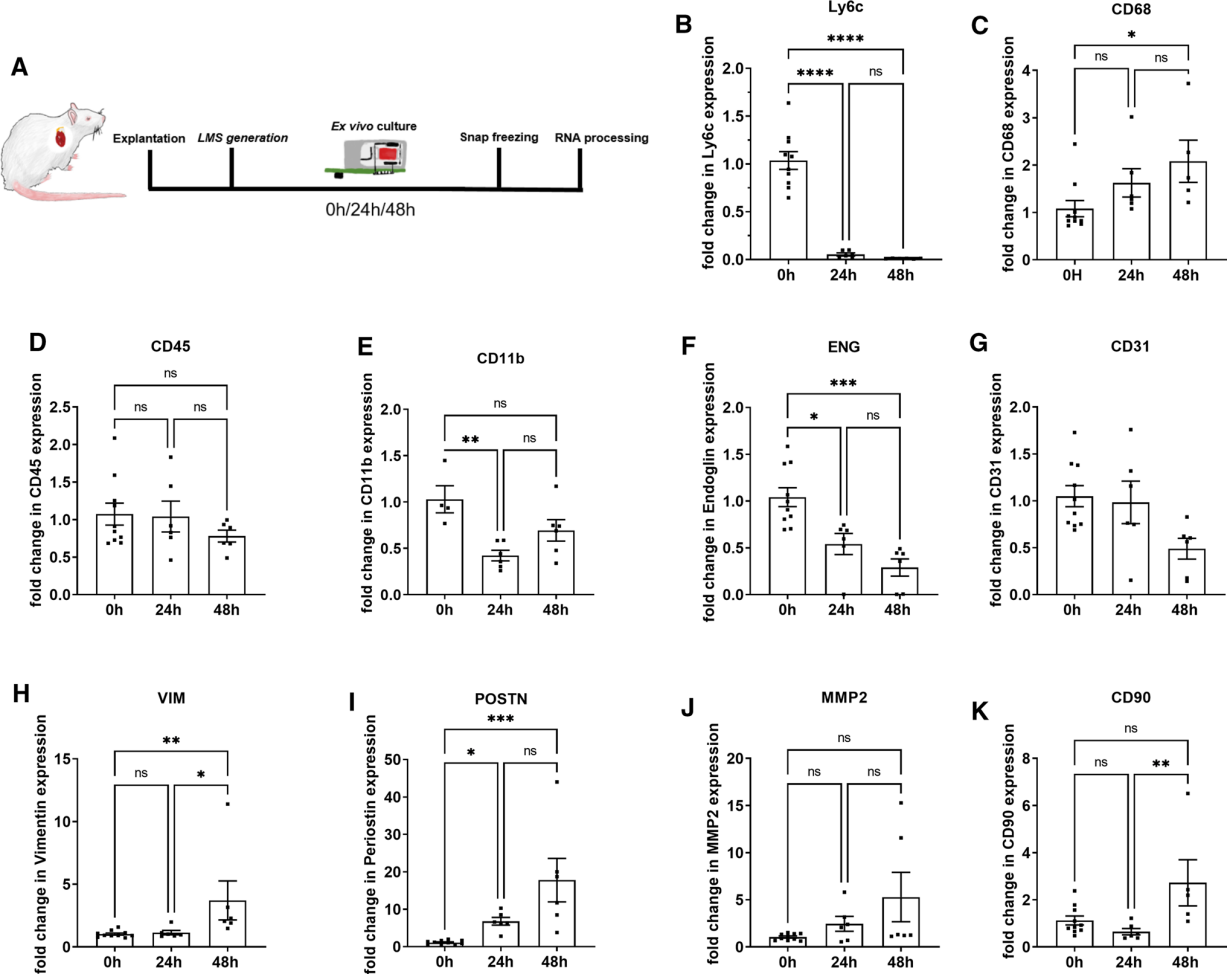
**A** Schematic of the experimental procedure. LMS were cultured ex vivo for 24 h and 48 h in biomimetic culture chambers and electrically paced at 1 Hz. The LMS were then snap-frozen in liquid nitrogen, and RNA was isolated for RT-PCR. **B**–**K**: Gene expression was normalized to the housekeeper genes *GAPDH* and *ACTB* (ΔΔ-CT). Inflammatory cell markers (*Lyc6*, *CD11b*, *CD68*, *CD45*; **B**–**E**) and endothelial markers (*ENG*, *CD31*; **F**–**G**) gene expression profiles were monitored over time. Gene expression of activated fibroblasts such as *VIM*, *POSTN*, *MMP2*, and *CD90* were also quantified (**H**–**K**). Prolonged ex vivo culture altered fibroblast, endothelial and lymphocyte-associated genes, but did not affect macrophage-associated genes. The experiment was repeated three times. 6–10 LMS were analyzed per group. ANOVA was performed with Turkey’s post hoc test or Kruskal–Wallis with Dunn’s post hoc test

Next, the LMS were prepared and biomimetically cultured ex vivo for 24 and 48 h to analyze time-dependent changes in gene expression profiles of various cell types (Fig. 2A). The RNA was isolated from freshly prepared slices and from 24 and 48 h ex vivo cultured slices and used to evaluate the gene expression profile of various cardiac cell population markers (Fig. 2B–K). *Ly6c* has been used in rat tissue to identify infiltrating cells [10]. The significant decrease in *Ly6c* expression, combined with the stable pan-macrophage gene marker *CD68*, confirmed that tissue macrophages were preserved during ex vivo culture despite the progressive withdrawal of infiltrating cells (Fig. 2B–C). *CD31* and *ENG* are adhesion molecules highly expressed by endothelial cells. The downregulation of *CD31* and *ENG* was expected due to the altered vascular homeostasis and the cessation of circulation in the capillaries (Fig. 2F–G). Although attenuated by the biomimetic culture, ex vivo culture conditions progressively activate cardiac fibroblasts [6, 36, 41], which transition to a myofibroblast phenotype [37]. In line with these observations, we also show the increased gene expression of various fibroblast genes such as *VIM*, *POSTN*, and *CD90*, suggesting a progressive activation of fibroblasts and their transition to a myofibroblast phenotype (Fig. 2H, I, K). These results confirmed that some cardiac cell types (mainly fibroblasts and endothelial cells) seem to undergo a degree of remodeling in the LMS following ex vivo culture and to indicate that the infiltrating cell number decreased over time.

**Fig. 3:**
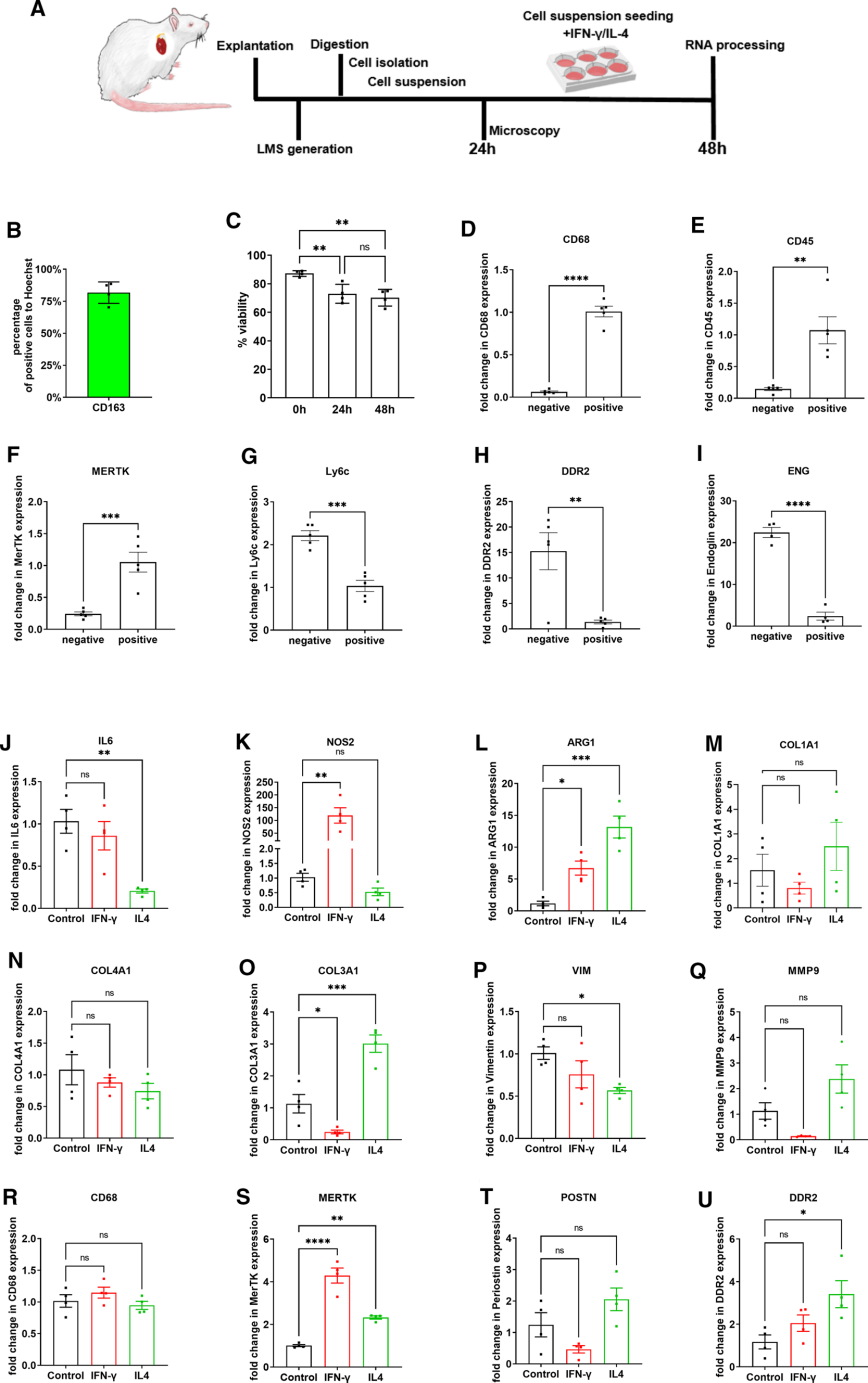
**A** Fresh LMS were digested with Collagenase A, and resident macrophages were isolated by MACS purification. **B** The cell suspension was separated with MACS CD11b/c beads and 1.5*105 cells of the positive fraction were seeded on gelatin-coated coverslips and fixated after 24 h in 4% PFA. The cells were then stained for CD163 resulting in a purity of 81.72% ± 8.39, mean ± SD. Three pictures per coverslip were averaged out of 4 separate isolations; 779 cells were counted in total. Full experiment with picture of the staining in Suppl. Fig. S1. **C** Cell viability at different time points of MACS purified rcMACs isolated from LMS was measured by dissociating the cells and counting their number with an automatic cell counter with 4% Trypan blue (mean ± SD) and for analysis an unpaired ANOVA with Turkey’s post hoc test was performed. **D**–**I** RNA was extracted from the positive and negative fractions of MACS CD11b/c separated cells of freshly digested LMS. Gene expression was analyzed by qPCR, expressed as ΔΔ-CT relative to *GAPDH* and normalized to the positive fraction. The two fractions were compared using an unpaired two-tailed Student’s *t*-test. The RNA was extracted out of 4 separate cell isolations performed from fresh LMS. **J**–**U** 1.5 × 10^5^ cells were cultured ex vivo and treated with recombinant rat 20 ng/ml IFN-γ (red) or 20 ng/ml IL-4 (green) for 48 h. Gene expression data were normalized to the untreated cell group. Unpaired ANOVA with Dunnett’s post hoc test was performed comparing the treatment groups to the control. Cells isolated from 4 hearts were analyzed

Next, we wanted to study if rcMACs isolated from LMS were responsive to chemical activation. RcMACs were isolated from freshly prepared LMS by MACS purification, seeded on coverslips and cultured for 24 or 48 h (Fig. 3A). Considering that CD11b/c is a marker that recognize not only macrophages, but also monocytes, dendritic cells, granulocytes, and NK cells [8, 31, 48], the efficiency and purity of the isolated cells were confirmed by immunostaining and RT-PCR. The adherent culture conditions also favored the removal of stromal cell types that prefer suspension cultures such as monocytes, dendritic and NK cells [35]. By immunostaining we showed that, after MACS purification, 91.4% of the total cells were *CD11b/c* positive and 81.7% were CD163 positive (Fig. 3B and Suppl. Fig. S1A). The qPCR data also indicated that *CD11b/c* + cells expressed lower *Ly6C* levels than the *CD11b/c-* population, and a lower expression level is regarded as typical for rcMACs (Fig. 3G) [47]. RT-PCR demonstrated that these rcMACs population presented higher gene expression for macrophage-specific genes such as pan-macrophage marker *CD68*, hemopoietic cell marker *CD45*, and *Proto-oncogene tyrosine-protein kinase MER* (*MerTK*) (Fig. 3D–F) than the negative population, which had higher levels of endothelial (*ENG*) and fibroblast specific genes (*DDR2*) (Fig. 3H, I). The isolated rcMACs population viability was measured after isolation at 0 h, 24 h and 48 h showing a reduction in viability to 70% after 48 h culture (Fig. 3C). To monitor the response of rcMACs to pharmacological stimulation, the isolated rcMACs were treated with either IFN-γ or IL-4 for 48 h (Fig. 3J–U). IFN-γ and IL-4 are immunomodulatory compounds reported to be on the opposite spectrum of the inflammatory activation process [38]. They are also commonly used to induce macrophages to acquire a pro- and anti-inflammatory phenotype [29]. The up-regulated gene expression, upon induction by IFN-γ, was linked to pro-inflammatory response, while these genes were down-regulated in IL-4 stimulated rcMACs. IFN-γ induced a dramatic increase (119.4-fold) in *NOS2* expression (Fig. 3K). *ARG1* was also up-regulated in the IFN-γ group (Fig. 3L). *MerTK* was also significantly up-regulated by 4.3-fold. IL-4 stimulation, on the other hand, significantly increased *MerTK* expression by 2.3-fold (Fig. 3S) and *ARG1* by 13.2-fold (Fig. 3L), while the expression of *IL-6* decreased (Fig. 3J). ECM-related genes were highly up-regulated in IL-4 stimulated rcMACs. *Col3a1* gene expression was also up-regulated (Fig. 3O), as well as collagen sensor receptor *DDR2* (Fig. 3U). *Vimentin* was significantly down-regulated in the IL-4-treated group (Fig. 3P). No differences were observed for *Col1a1*, *Col4a1*, *CD68*, *MMP9* and *POSTN*. These data demonstrate that the MACS isolated rcMAC can respond to the ex vivo stimulation acquiring a pro- or anti-inflammatory phenotype.

**Fig. 4:**
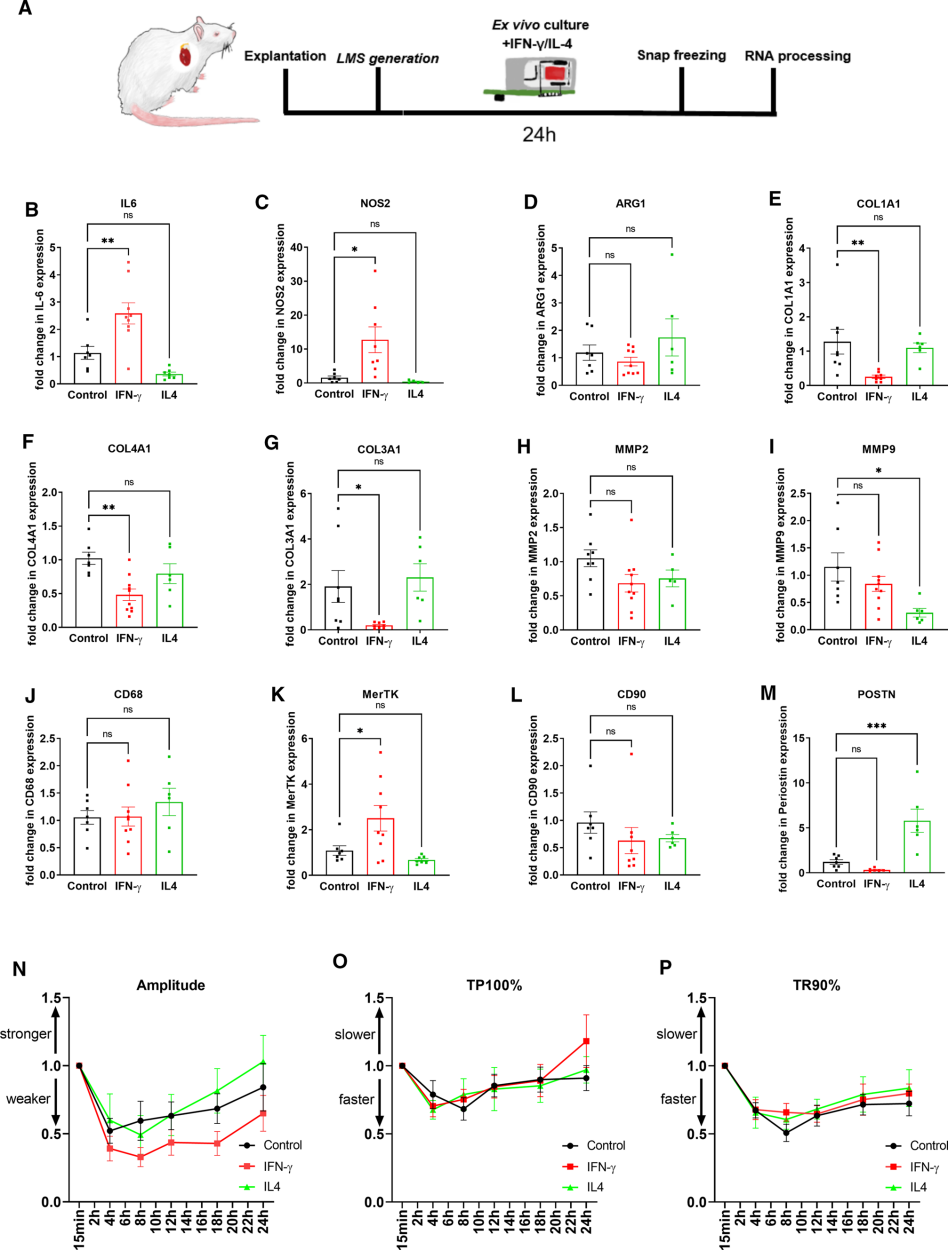
**A** LMS were cultured for 24 h and treated with recombinant rat 20 ng/ml IFN-γ or 20 ng/ml IL-4. **B**–**M** Gene expression was analyzed by RT-PCR and expressed as ΔΔ-CT relative to two housekeeper genes (*GAPDH* and *ACTB*). The data were normalized to the untreated LMS group. Normally distributed samples were compared by unpaired ANOVA with Dunnett’s post hoc test, not normally distributed samples with a Kruskal–Wallis test with Dunn’s post hoc test. 6–9 LMS from 5 animals were analyzed. **N**–**P** Tissue contraction kinetics of LMS cultured for 24 h were unchanged in LMS treated with recombinant rat 20 ng/ml IFN-γ or 20 ng/ml IL-4. Contraction amplitude, time to peak 100%, and 90% of relaxation time were measured. The data were normalized to the earliest time point. In the control group, 14 recordings were analyzed compared to 12 IFN-γ-treated and five IL-4-treated LMS

Multicellularity is essential in tissue biology, and the interaction between different cell types dramatically affects their response to various chemical and mechanical stimuli. To investigate the reaction of the myocardium to pro- and anti-inflammatory stimulations, LMS were treated with the immunomodulatory compounds IL-4 and IFN-γ for 24 h (Fig. 4A). The LMS were snap-frozen in liquid nitrogen, and the RNA was extracted for RT-PCR. The 24 h treatment with IFN-γ increased the *IL-6* gene expression profile by 2.6-fold (Fig. 4B) and *NOS2* by 12.7-fold (Fig. 4C). All three collagen genes (*Col1A1*, *Col3A1*, *Col4A1*) were significantly down-regulated in the rat IFN-γ-treated group (Fig. 4E–G). *MerTK* expression was also increased compared to the control group (Fig. 4K). The inflammatory stimulation induced by IL-4 did not cause such a dramatic change in the LMS gene expression profile. The only altered genes were *POSTN* (Fig. 4M) and the extracellular matrix-remodeling gene *MMP9*, which were down-regulated (Fig. 4I).

The biomimetic culture device used for the ex vivo culture of LMS allows a constant recording of the LMS contractility. Considering the altered gene expression of various inflammatory, collagen, and fibrosis-related genes, we investigated the contraction parameters of the treated LMS (Fig. 4N–P). Force generation and contraction kinetics (time to peak and relaxation time (T90), and decay rate) were unchanged between the three groups suggesting that the tissue contraction was maintained in all the groups and was not affected by the pharmacological stimulation.

Overall, our results suggest that the LMS responded to both pro- and anti-inflammatory stimulations with no change of the contraction kinetics. However, the multicellular environment seems to reduce the level of activation when compared to cells cultured on plastic substrates. Finally, IFN-γ is capable of inducing a stronger inflammation state in the LMS compared to IL-4.

**Fig. 5:**
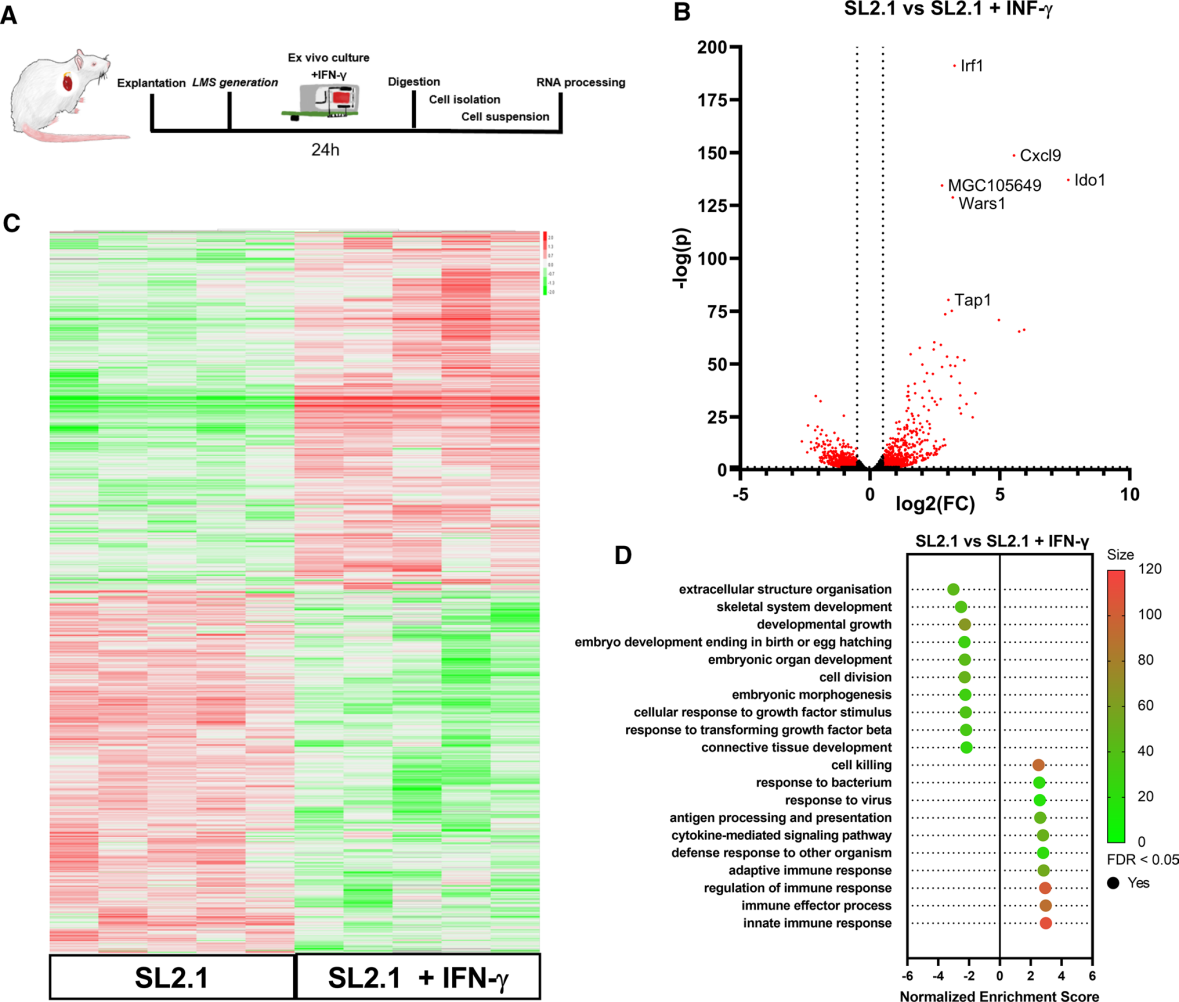
**A** LMS were cultured for 24 h and treated with recombinant rat 20 ng/ml IFN-γ. Afterwards, the LMS were digested, and rcMACs were isolated by MACS purification. Low Input RNA-seq was performed with 1 ng per sample. **B** Vulcan plot of 12,935 genes, compared between SL2.1 vs SL2.1 + IFN-γ isolated cells, − log(*p*) of FDR < 0.05 adjusted *p* values and log2(FC). 1511 genes (red-marked dots) are − log(*p*) > 1.301 and │log(FC)│ > 0.5. **C** Heatmap of RNA-seq results (DESeq2) comparing SL2.1 vs SL2.1 + rat IFN-γ isolated cells *n* = 5 animals per group. **D** TOP10 GO Term (biological process) enrichment analysis (GSEA) of the significantly deregulated genes. *X*-axis: Normalized enrichment score SL2.1 vs SL2.1 + IFN-γ. A positive score indicates enrichment in the rat IFN-γ stimulated cells. *Y*-axis: GO-term pathway names sorted from least to most enriched. The color grading of the dots specifies the percentage of enriched genes matching the GO-gene set. A large dot size specifies significant FDR < 0.05

Both cardiomyocytes and fibroblasts respond to IFN-γ stimulation [22, 39]. After assessing the global changes of the pharmacological stimulation occurring in the cultured LMS (Fig. 4), which provided the overall response occurring at a tissue level, and therefore from all the various cardiac cells, we investigated the single rcMAC population. Briefly, the LMS were cultured with 20 ng/ml of rat IFN-γ for 24 h, digested with collagenase and the rcMACs were isolated by MACS purification (Fig. 5A). The transcriptomic gene expression profile of the isolated cells is shown in the heatmap (Fig. 5C). After plotting 12,935 genes with a base mean above 5 in a Volcano plot, red-marked genes were significantly and relevantly down-regulated (− log(*p*) < 1.301 and │log(FC)│ > 0.5, 1511 genes) (Fig. 5B). The down-regulated genes in treated rcMACs such as *NOS2* (log(FC) 1,41/− log(*p*) 11,26,896), *MerTK* (log(FC) 1,23/− log(p) 14,67) and *COL3A1* (log(FC) − 1,80/− log(*p*) 13,24) (data extracted from RNA-seq data set) show a similar pattern to our RNA-seq result of ex vivo cultured rcMACs (Fig. 3K, O, S). The analysis identified that the ten most significantly up-regulated genes were *IRF1*, *CXCL9*, *IDO1*, *MGC105649*, *Wars1*, *Tap1*, *Timp3*, *Stat1*, *Batf2* and *Ifgga4*. The ten most significantly deregulated genes were *Col6a1*, *Igsf10*, *Dchs1*, *Fxyd6*, *Col5a1*, *Col6a2*, *Serpinf1*, *C1orf115*, *Tnfaip6* and *Col5a3*. Using GO Term (biological process) enrichment analysis (GSEA) of the significantly changed genes, we were able to identify several significantly down-regulated pathways (Fig. 5D), which included immune effector response, process and regulation, cytokine-mediated signaling and response to a virus. This data indicated that LMS treatment with IFN-γ clearly shifted the endogenous rcMACs toward a pro-inflammatory phenotype.

**Fig. 6:**
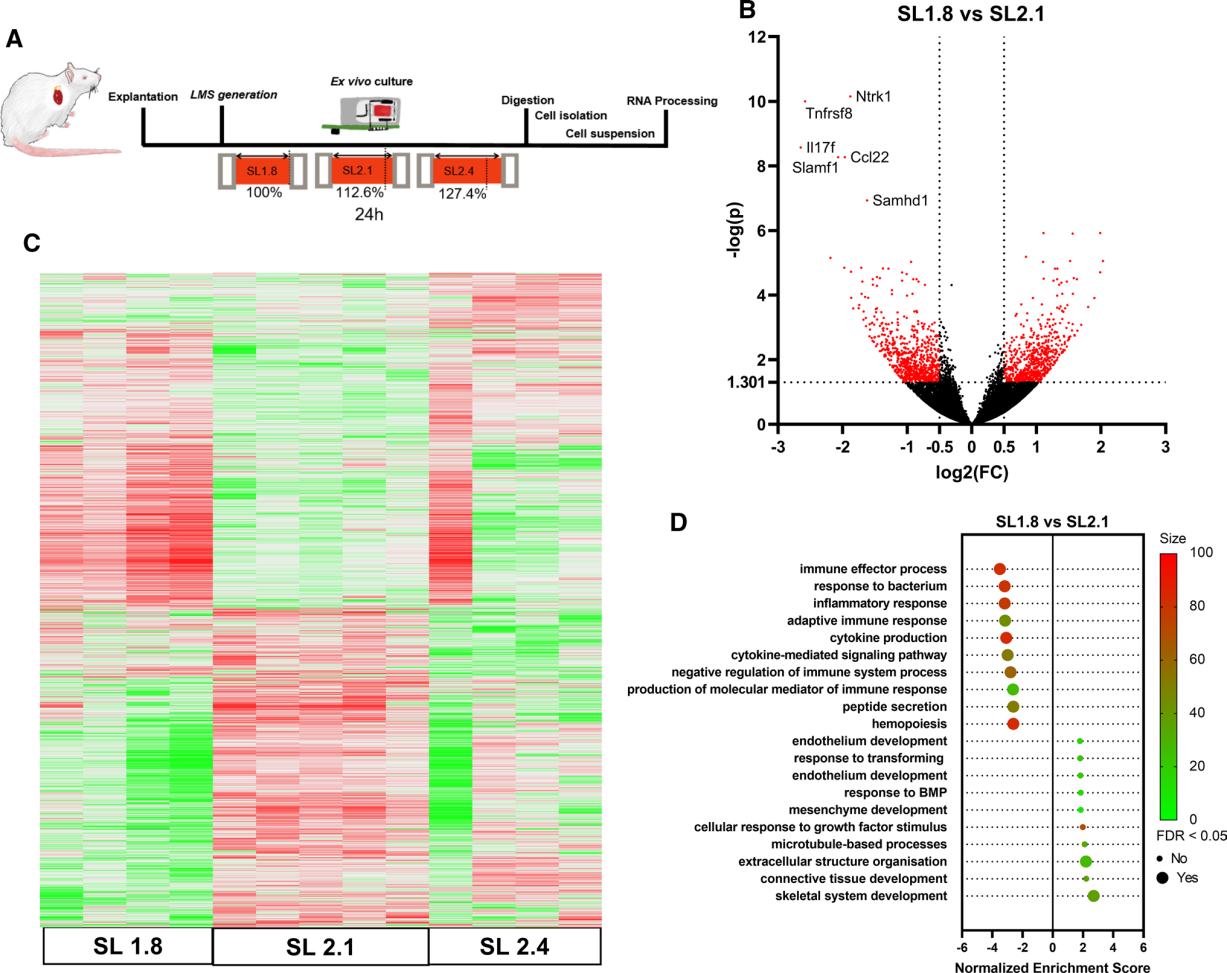
**A** LMS were cultured for 24 h, and the LMS were stretched to apply an initial preload that corresponded to SL1.8 (unloaded), SL2.1 (physiological load) and SL2.4 (high load). Afterwards, the tissue was digested, and MACS-facilitated cell separation was performed to isolate the rcMACs. Low-Input RNA-Seq was performed with 1 ng per sample. **B** Vulcan plot of subgroup SL2.1 vs SL1.8 with genes. *X*-axis: log2(FC) *Y*-axis: − log(*p*) of FDR < 0.05 adjusted *p*-values. 1428 genes (red-marked dots) are − log(p) > 1.301 and Ilog(FC)I > 0.5. **C** Heatmap of RNA-Seq results (DeSeq2) comparing SL1.8 vs SL2.4 vs SL2.1 of isolated cells *n* = 4–5 isolations per group. **D** TOP10 GO Term (biological process) enrichment analysis (GSEA) of the significantly deregulated genes. *X*-axis: Normalized enrichment score SL1.8 vs SL2.1. A negative score indicates enrichment in the cells after unloading. *Y*-axis: GO-term pathway names sorted from least to most enriched. The color grading of the dots specifies the percentage of enriched genes matching the GO-gene set. A large dot size specifies significant FDR < 0.05

Alterations of the cardiac contraction and the forces experienced by the various cell types are known to alter cellular function [13, 50, 52]. Conditions of unload or overload can influence cardiomyocytes contractile properties with altered calcium transients [2]. Fibroblasts also respond by transitioning to a myofibroblast phenotype and increased collagen deposition [33]. Previous studies have demonstrated that modifications of the preload can be applied and investigated in LMS [43, 57]. However, those studies have only examined the global transcriptome changes originating from all the cardiac cells that make up the LMS [57]. Here, we investigated the effect of preload on phenotype changes of rcMACs. To do so, the initial preload of the LMS was set to obtain a sarcomere length (SL) of 1.8 (unloaded; no stretch applied—0%), 2.1 (physiological load; 12.6% of stretch applied) or 2.4 (high load; 26.36% of stretch applied). After 24 h on biomimetic ex vivo culture, rcMACs were isolated (Fig. 6A). The heatmap (Fig. 6C) demonstrates the transcriptomic alteration of rcMACs during mechanical changes. When SL1.8 vs SL2.1 were compared in the Vulcan plot, 1428 out of 13,077 plotted genes were significantly deregulated (− log(*p*) < 1.301 and │log(FC)│ > 0.5) (Fig. 6C). The ten most significantly up-regulated genes were *Acta2*, *Plau*, *Col12a1*, *Tuba1a*, *Lypd1*, *C1qtnf6*, *Sigmar1*, *Adamts9*, *Tpm2,* and *Efemp1*. The ten most significantly deregulated genes were *Ntrk1*, *Tnfrsf8*, *Il17f*, *Slamf1*, *Ccl22*, *Samhd1*, *Cerkl*, *Slfn13*, *Il10,* and *Cpa3*. GO Term (biological process) enrichment analysis (GSEA) identified various altered pathways, which included immune effector processes, inflammatory response cytokine production and cytokine production (Fig. 6D).

**Fig. 7:**
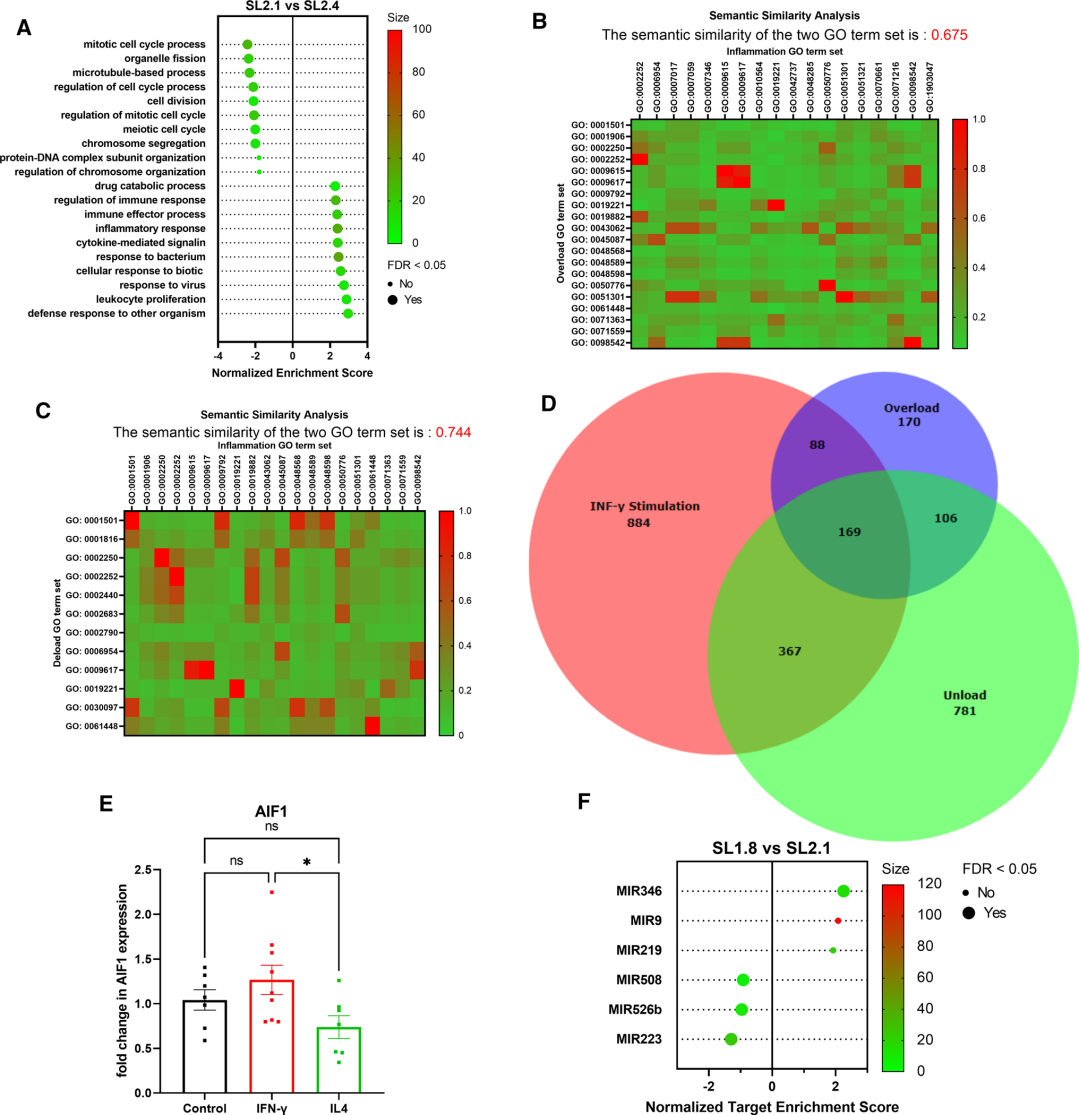
**A** TOP10 GO Term (biological process) enrichment analysis (GSEA) of the significantly deregulated genes of SL2.1 vs SL2.4. *X*-axis: Normalized enrichment score SL2.1 vs SL2.4. A positive score indicates enrichment in the cells with the overload condition. *Y*-Axis: GO-term pathway names sorted from least to most enriched. The color grading of the dots specifies the size of enriched genes matching the GO-gene set. A large dot size specifies if significant FDR < 0.05. **B** Heatmap of the semantic similarity analysis of the TOP 10 GO-term GSEA results of cells influenced by inflammatory stimulus or LMS load change ranked decreasingly by number. Effects can range from 0 (no similarity; green) to 1 (high similarity; red). The overall semantic similarity is 0.675, indicating an intermediate semantic similarity between the two sets. **C** Heatmap of the semantic similarity analysis of the TOP 10 GO-term GSEA results of cells influenced by inflammatory stimulus or LMS load change ranked increasingly by number. The overall semantic similarity is 0.744, indicating an intermediate semantic similarity between the two sets. **D** Proportional size Venn diagram of the significantly deregulated genes of IFN-γ stimulated, unloaded and overload rcMACs. **E** LMS were cultured for 24 h and treated with recombinant rat 20 ng/ml IFN-γ or 20 ng/ml IL-4. Gene expression was analyzed using SYBR Green-based RT-PCR and expressed as ΔΔ-CT relative to the housekeeper gene *ACTB*. The data were normalized to the untreated LMS group. Unpaired ANOVA with Turkey’s post hoc test was performed comparing the groups. 6–9 LMS from five animals were analyzed. **F** In silico GESEA network prediction (TOP10/ affinity propagation clustered to TOP6) of miRNAs targets for rcMACs cultured at SL1.8 vs SL2.1 based on the significantly deregulated genes regardless of log(FC) change. *X*-axis: Normalized target enrichment score. A positive score indicates enrichment of the target at physiological load (SL2.1). *Y*-axis: miRNA names sorted from most to least enriched. Color grading of the dots specifies the size of enriched genes matching the miRNA set. A large dot size specifies, if significant FDR < 0.05

When the LMS was subjected to a high preload (SL2.4), the most significantly altered pathways included leukocyte proliferation, increased defense response to other organisms, inflammatory response and cytokine-mediated signaling. After performing GSEA analysis, we compared the resulting GO-term sets using statistical semantic similarity analysis (Fig. 7B). This test scores similarity between 0 and 1, no similarity and high similarity, respectively. The analysis revealed an intermediate similarity of 0.657 between the GO-term sets of IFN-γ and overload influenced rcMACs. We compared the fractions of CD11b/c and CD163 positive cells from LMS cultivated for 24 h at SL2.1 and incubated with IFN-γ vs LMS with SL1.8. The LMS were fixated and stained for CD11b/c and CD163, VIM and Hoechst33342. Analysis showed significantly more VIM-positive cells in the SL2.1 + IFN-γ group (*p* ≤ 0.05), but no changes in the total numbers of CD11b/c or CD163 cells (Suppl. Fig. S2A) or the fraction of CD163/CD11b/c cells (Suppl. Fig. S2B). Our results indicate that rcMACs respond to the increased preload similar as when activated by a chemical stimulus such as IFN-γ, with intermediate similarity.

To conclude our study and understand whether unload had a similar effect to IFN-γ stimulation, we performed a semantic similarity analysis between the two groups (Fig. 7C). The data revealed an intermediate semantic similarity of 0.744 between IFN-γ and mechanical unloading on rcMACs. The mapping of the significantly deregulated genes resulted in the overlap of 376 shared genes (Fig. 7D). One of the genes in the overlap between unloading and IFN-γ stimulation is *Allograft inflammatory factor 1* (*AIF-1*). *AIF-1* is expressed by macrophages and was first reported in rat cardiac allografts with chronic immune reactions [53]. By being highly expressed in macrophages, whole LMS could confirm differences in expression. RT-PCR confirmed the significant deregulation of the *ALF-1* gene in immunomodulatory treated LMS (Fig. 7E). Using GSEA analysis to identify miRNA targets in the treated groups (SL1.8 vs SL2.1), we identified four miRNAs (miRNA-346, miRNA-508, miRNA-526b and miRNA-223) (Fig. 7F). Mi-RNA 346 is an enriched target of the SL2.1 transcriptome changes in rcMACs. Mi-RNA 508, miRNA-526b and miRNA-223 are associated with unloaded rcMACs (Fig. 7F). To summarize, ex vivo culture in an unloaded state induced a response in the isolated rcMACs similar to their response to IFN-γ treatment. We also identified four potential miRNA targets which could be verified and tested to develop new therapies*.*

## Discussion

In this study, LMS were used for the first time to investigate the effects of chemical and mechanical chronic stimulation on a specific immune cardiac cell type, the rcMACs, and their response when cultured in the multicellular physiological environment of the myocardium. This approach has the substantial advantage of being multicellular and disconnected from circulation, hereby excluding infiltrating immune cells. It provides a unique research tool to investigate this specific and rare, but essential resident cell type. Our major findings were:

1. The immunomodulatory stimulation influenced the overall gene expression profile without altering tissue contraction.
2. RcMACs could be reliably isolated from LMS treated with mechanical or chemical stimuli and investigated for transcriptome alterations. Tissue unloading induced rcMACs to acquire a phenotype similar to pro-inflammatory chemical stimulation. Overloading also caused a pro-inflammatory phenotype, but different gene sets were involved.
3. In silico analysis predicted miRNA targets that could be used for future therapeutic approaches explicitly targeting rcMACs.

In this study, rcMACs were immunostained with various antibodies, including VIM, CD45 and CD163. VIM is a commonly used marker to identify stromal cells; the combination of VIM with the immune markers CD45 and CD163 was used to identify and count the number of rcMACs and leukocytes relative to the stromal cells. CD163 is an immune cell marker exclusively expressed by rat tissue macrophages [45]. In line with the literature, CD163 + cells, corresponding to the rcMACs, were 7.6% of the total stromal cell population [12]. This study performed rcMAC counts by counting cells after images were acquired with confocal microscopy. Flow cytometry or Cre/loxP reporter lineage tracking are alternatives to this approach. However, although laborious and time-consuming, this approach is simple and effective, as was previously used to quantify other cell types [41]. The imaging and quantification are also limited by antibody penetration depth and, therefore, restricted to the LMS surface.

The LMS contractility and gene expression profile quantification confirmed overall high viability and preserved preparation functionality over the 24 h of ex vivo biomimetic culture. As previously reported [6, 57], we also observed some degree of adaptation to the new culture conditions with increased expression of some genes associated with fibroblasts activation, such as *Periostin* and *Vimentin* or endothelial cell de-differentiation (*CD31* and *ENG*). The supplementation of humoral stimuli and metabolic substrates to the culture media has been reported to favor the needs of the myocardium in culture and to delay the onset of cell de-differentiation [36]. The LMS were cultured in a serum-free media as recommended in several publications [6, 57] and to be less dependent on batch to batch serum content variability. The most noticeable data were the dramatic decline of Ly6c, which suggest the rapid depletion of the cardiac granulocytes. Notably, CD68 levels were maintained, meaning, together with the confocal quantification, that the rcMACs can survive the ex vivo culture. Rat myocardial slices were used in this study. It has repeatedly been reported that rat tissue undergoes a faster tissue adaptation and remodeling than LMS prepared from larger animal models [6, 41]. These models could provide a more translational aspect to this study and allow for more prolonged ex vivo chronic treatment. These further studies would be relevant to further monitor and understand the role of rcMACs during chronic stimulation.

This study successfully isolated resident macrophages from both cardiac tissue and cardiac tissue slices after 0 h and 24 h using magnetically labeled antigen facilitated cell separation (MACS). This method is a reliable and straightforward approach to isolate specific cardiac populations like macrophages [4]. CD11b/c is present on macrophages and granulocytes [48] and is currently the only available Miletenyi Biotec MACS antibody for rat macrophages, limiting the specific isolation of rcMACs cell population. An alternative to this approach is Fluorescence Activated Cell Sorting (FACS) sorting, combining more antibodies and isolating and analyzing smaller or more specific cardiac sub-populations. The expression of CD11b was significantly reduced after 24 h, which can be caused by cell depletion or cell de-differentiation. Overall, the amount of RNA after isolation was low, but sufficient to be used for Low-input RNA-Seq. This limited the viable post experiment analysis options.

In this study, IFN-γ and IL-4 were chosen as chemical stimuli to activate rcMACs when cultured ex vivo or in the multicellular environment of the LMS. These two cytokines have extensively been used both ex vivo and in vivo [24, 46, 59] to induce a pro- and anti-inflammatory state in the macrophages. However, their effects exclusively on the rcMAC population have never been tested. Our data demonstrate that they are potent activators of rcMACs ex vivo. Mechanical forces are also potent regulators of cell behavior and inter-cellular communication. Alterations to cardiac tissue contraction, such as unloading or overloading conditions, are known to affect both cardiomyocytes and cardiac stromal cells [43]. In such pathological states, cardiac fibroblasts transition to an activated state, and they secrete and deposit interstitial collagen contributing to tissue fibrosis [60]. Macrophage role in tissue fibrosis is still unclear and under intensive investigation. Even less is known about rcMACs and their response to mechanical stimuli. In this study, we investigated and compared both the effects of chemical and mechanical activation of this specific cell population. As the number of rcMACs in the steady state is under ten percent of all stromal cells, marker genes of rcMAC activation measurable in the whole tissue should be identified to ease further investigation. Comparing clusters of deregulated genes, we identified *AIF-1* as a possible marker gene of rcMACs after overlapping inflammation and unloading transcriptomic results. It is known that *AIF-1* is induced by IFN-γ stimulation and plays a role in warm and cold ischemia–reperfusion injury in the rat liver [20] or is expressed by macrophages in injured skeletal muscles [23]. We now add to the knowledge that *AIF-1* may be involved as a marker of inflammation in the unloaded heart.

MicroRNAs are novel and potent treatment options for cardiovascular disease and cardiomyocyte regeneration [1] and can be used as potential biomarkers [11]. RcMACs could be potential therapeutic targets for miRNAs in the heart [48]. MicroRNA-223 was identified as a target in rcMACs affected by unloading the LMS. It has already been reported that MicroRNA-223 is associated with pro-inflammatory macrophage polarization in murine macrophages [5]. MicroRNA-346, on the other hand, has not been associated with resident cardiac macrophages yet and could therefore be an exciting novel target. These newly identified microRNAs will indeed require further ex vivo and in vivo testing to evaluate and understand their effectiveness for therapeutic use.

## Conclusion

RcMACs were studied ex vivo during prolonged tissue culture in their native environment for the first time. This approach also avoided circulating cell infiltration providing a platform to explore this rare but physiologically relevant cell type. We moreover demonstrated for the first time that a specific cell population can be isolated from LMS post ex vivo culture. Our work shows the ability to change culture conditions mimicking inflammation or alterations of the preload, which induced a similar activation of the rcMACs. In addition, we used low input transcriptome analysis to investigate deregulated inflammatory pathways leading to a new application of marker genes and identification of miRNA targets that could be used for future therapies. Our observations support the use of LMS as a platform to better identify rcMAC regulation and to study rcMACs-associated cardiac inflammation and fibrosis mechanisms. LMS can assist in finding new possible treatment options in translational discovery processes.

## Supplementary Information

Below is the link to the electronic supplementary material.

Figure S1. **A** Fresh LMS were digested with Collagenase A, and resident macrophages were isolated by MACS purification. The cell suspension was separated with MACS CD11b/c beads and 1.5*105 cells of the positive fraction seeded on gelatin-coated coverslips and fixed after 24 h in 4% PFA. The cells were then stained for CD163 and CD11b/c and imaged by fluorescence microscopy. 91.36% ± 3.54 (mean ± SD) and 81.72% ± 8.385 (mean ± SD) of the cells were respectively CD11b/c or CD163 positive with a ratio of 89.31% ± 6.827 (mean ± SD) CD163/CD11b/c. Three pictures per coverslip were averaged out of four separate isolations; 779 cells were counted in total. **B** Representative pictures of isolated cells stained with Hoechst33342 (blue), CD11b/c (red) and CD163 (green) (TIF 6205 KB)

Figure S2. LMS were cultured ex vivo for 24h in biomimetic culture chambers and electrically paced at 1Hz. To compare the effect of IFN-γ or unloading on macrophage cell number, LMS were either cultured unloaded or with recombinant rat 20 ng/ml IFN-γ. They were stained with antibodies (VIM/CD11b/CD163) and imaged using a confocal microscope. **A** The quantification revealed significantly more VIM-stained cells in the SL2.1 + IFN-γ group (p<0.05) but no change in the total number of CD11b/c or CD163 cells. **B** The amount of quantified CD163 cells was divided by the amount of CD11b/c cells. There was no significant change in cell ratio. **C** Representative average Z-stack projected pictures of LMS stained with Hoechst33342 (blue), VIM (red), CD11b/c (yellow) and CD163 (green). 1-2 pictures from three myocardial slice sections produced from three animals were processed per group, and 2906 nuclei were counted in total (TIF 23276 KB)

## Data Availability

The analyzed datasets are available from the corresponding author upon request.
