# Supplementary figures and images for: Chemical and mechanical activation of resident cardiac macrophages in the living myocardial slice ex vivo model
Source: Basic Res Cardiol. 2022 Nov 30;117(1):63. doi: 10.1007/s00395-022-00971-2 (PMC9712328; doi:10.1007/s00395-022-00971-2)

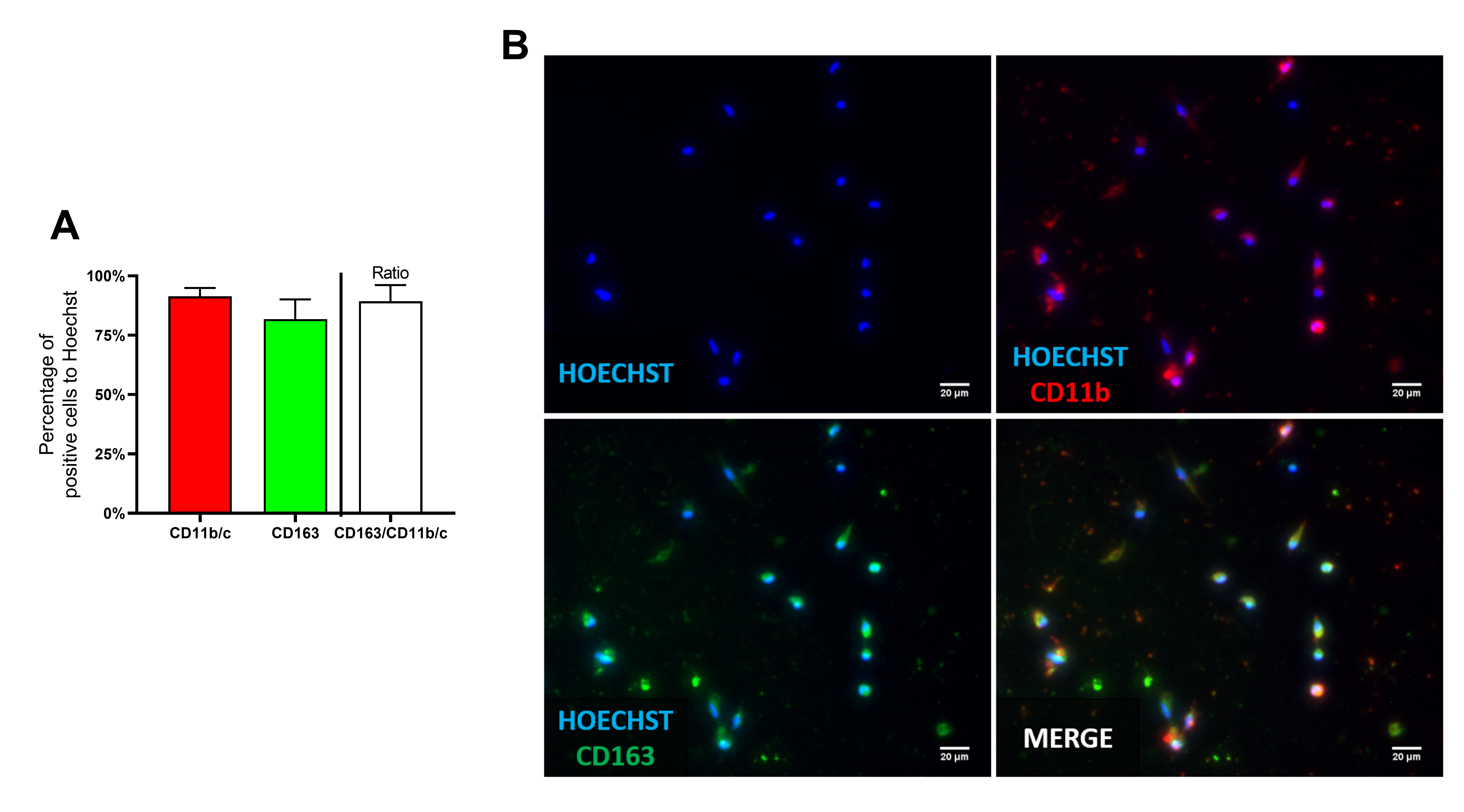

Supplement: Supplementary file 1 — Figure S1. A Fresh LMS were digested with Collagenase A, and resident macrophages were isolated by MACS purification. The cell suspension was separated with MACS CD11b/c beads and 1.5*105 cells of the positive fraction seeded on gelatin-coated coverslips and fixed after 24 h in 4% PFA. The cells were then stained for CD163 and CD11b/c and imaged by fluorescence microscopy. 91.36% ± 3.54 (mean ± SD) and 81.72% ± 8.385 (mean ± SD) of the cells were respectively CD11b/c or CD163 positive with a ratio of 89.31% ± 6.827 (mean ± SD) CD163/CD11b/c. Three pictures per coverslip were averaged out of four separate isolations; 779 cells were counted in total. B Representative pictures of isolated cells stained with Hoechst33342 (blue), CD11b/c (red) and CD163 (green) (TIF 6205 KB) [file 395_2022_971_MOESM1_ESM.tif]

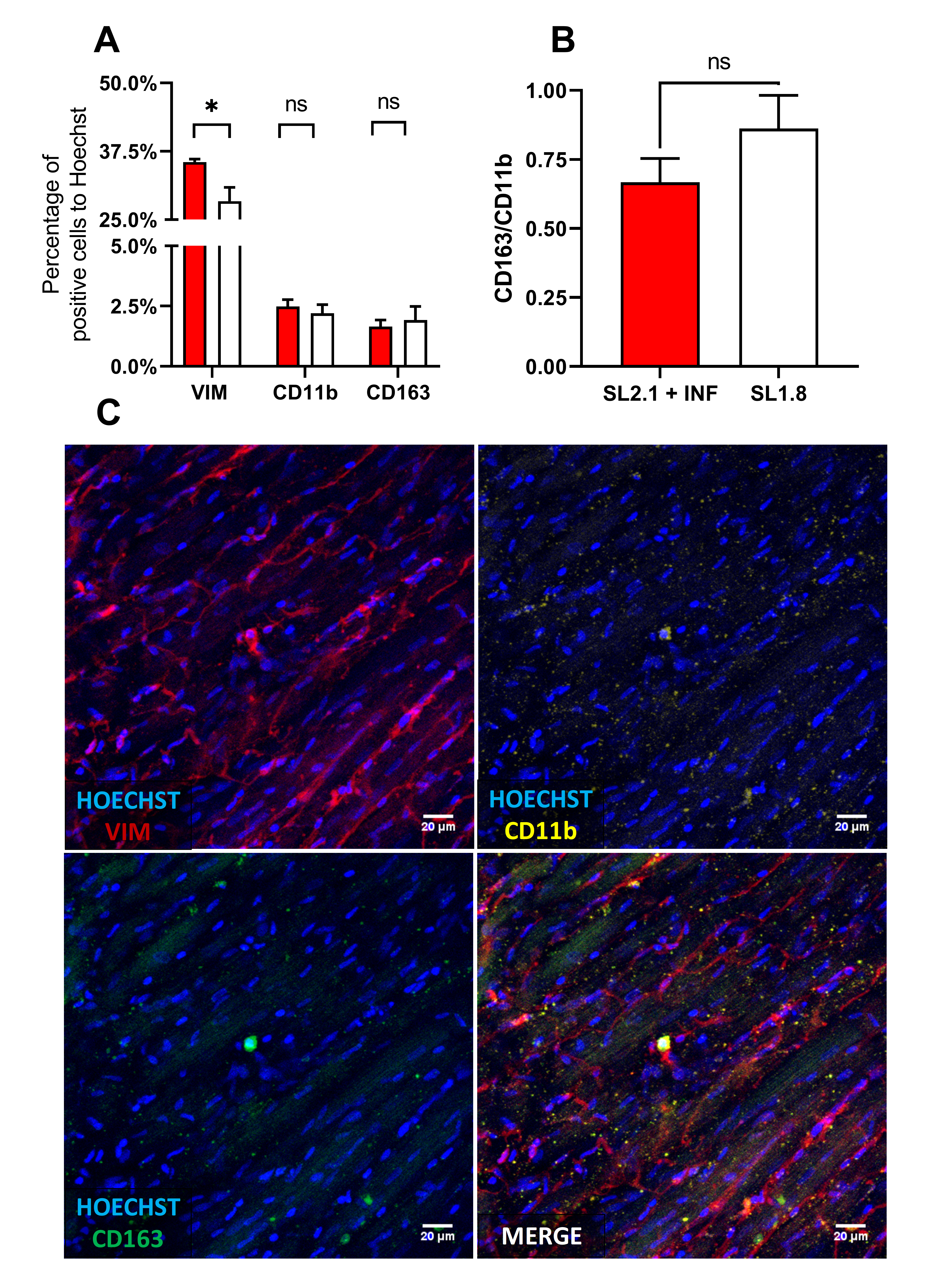

Supplement: Supplementary file 2 — Figure S2. LMS were cultured ex vivo for 24h in biomimetic culture chambers and electrically paced at 1Hz. To compare the effect of IFN-γ or unloading on macrophage cell number, LMS were either cultured unloaded or with recombinant rat 20 ng/ml IFN-γ. They were stained with antibodies (VIM/CD11b/CD163) and imaged using a confocal microscope. A The quantification revealed significantly more VIM-stained cells in the SL2.1 + IFN-γ group (p<0.05) but no change in the total number of CD11b/c or CD163 cells. B The amount of quantified CD163 cells was divided by the amount of CD11b/c cells. There was no significant change in cell ratio. C Representative average Z-stack projected pictures of LMS stained with Hoechst33342 (blue), VIM (red), CD11b/c (yellow) and CD163 (green). 1-2 pictures from three myocardial slice sections produced from three animals were processed per group, and 2906 nuclei were counted in total (TIF 23276 KB) [file 395_2022_971_MOESM2_ESM.tif]
